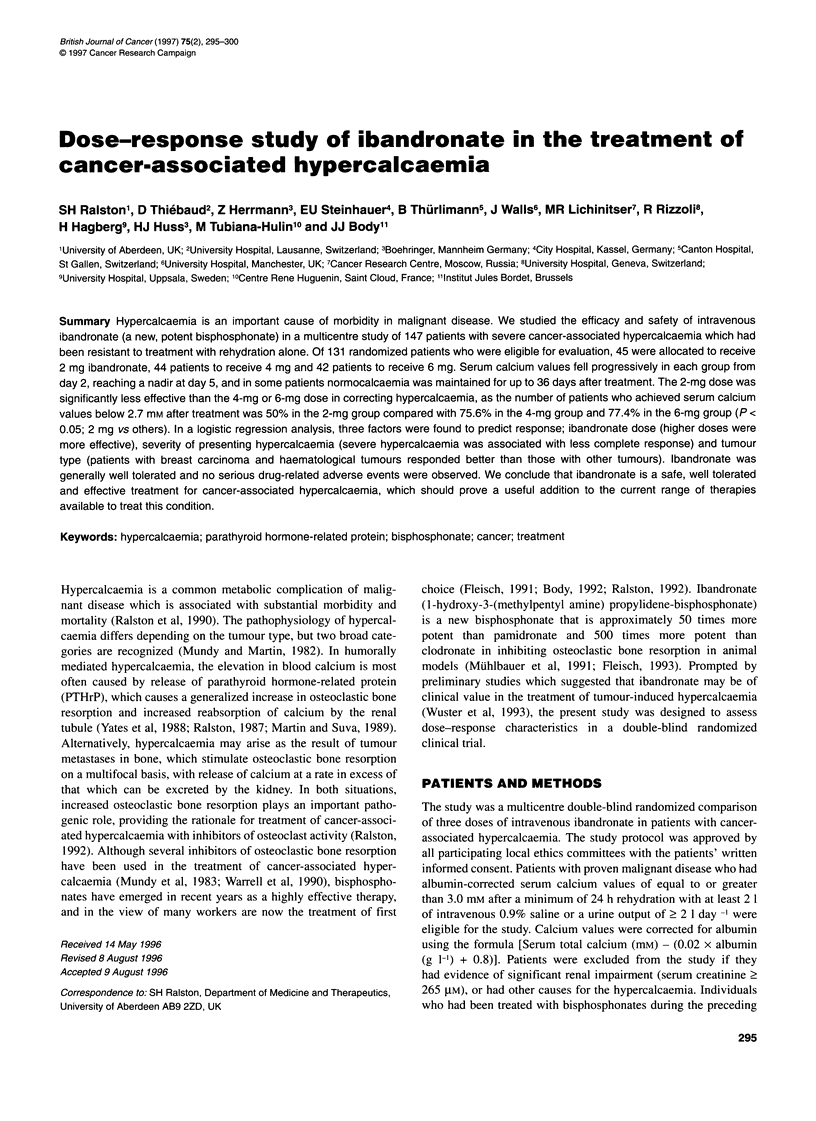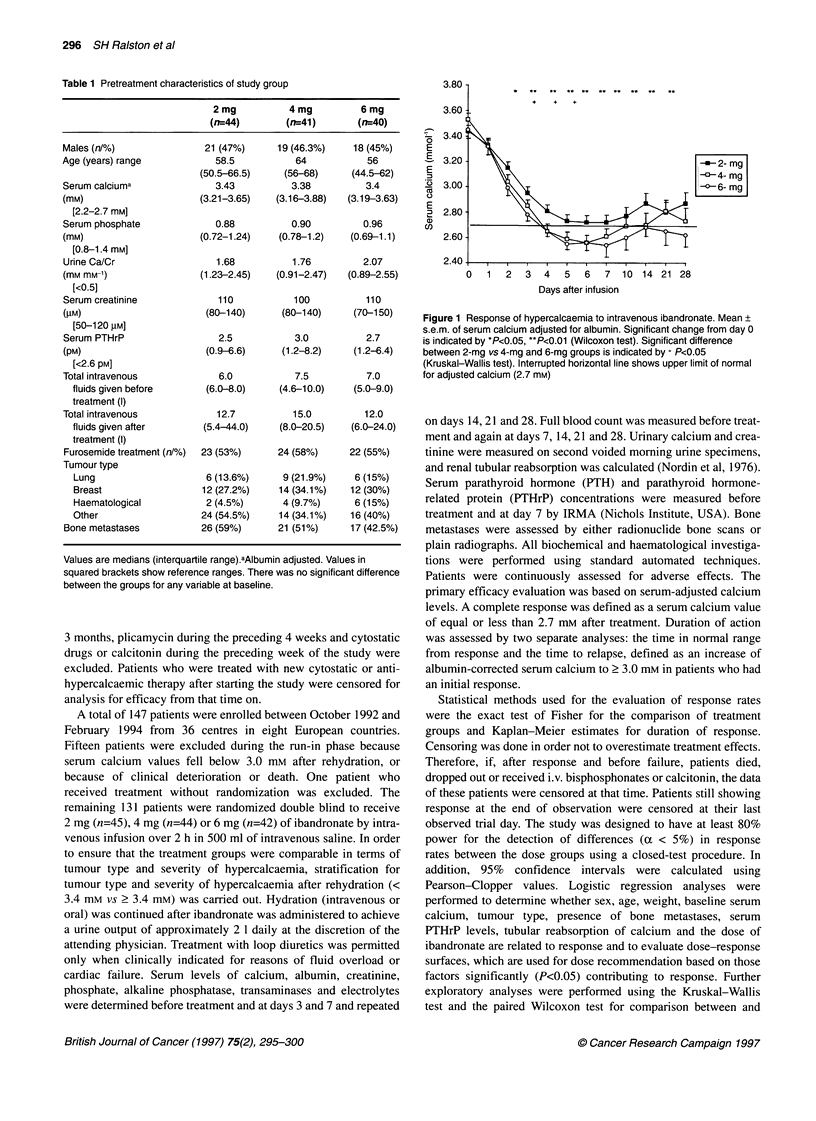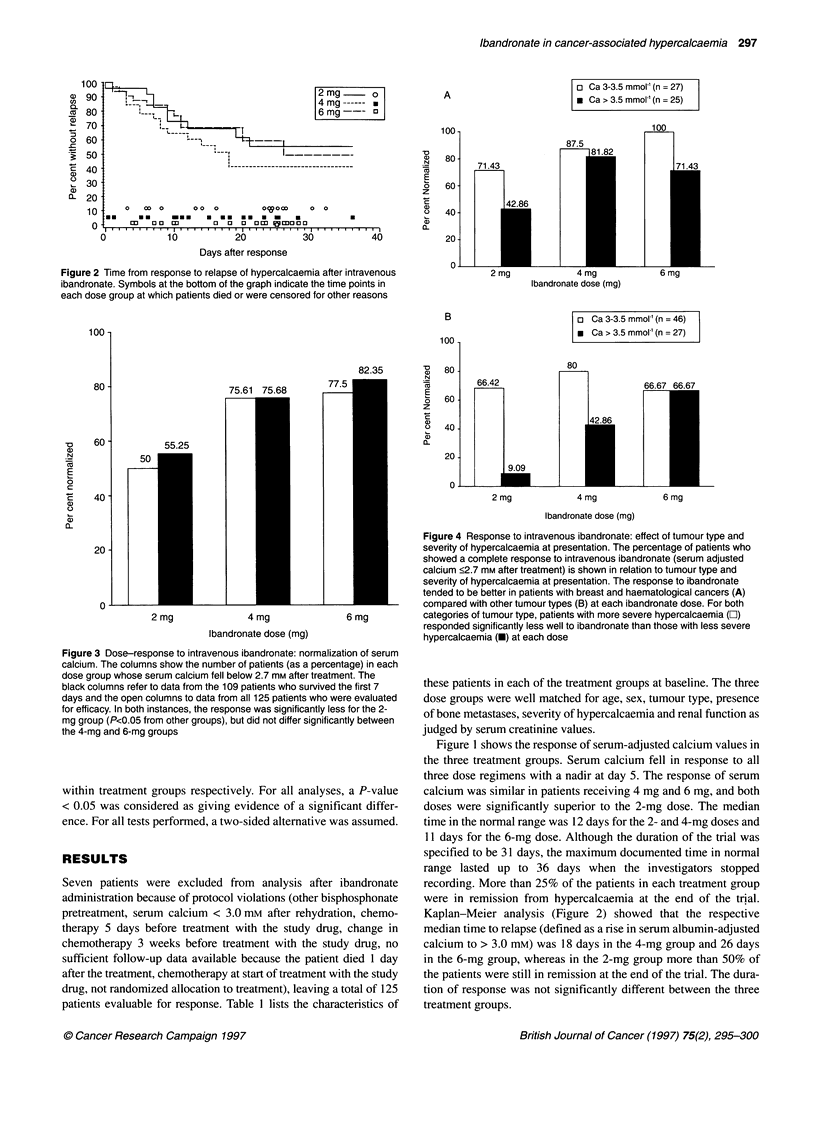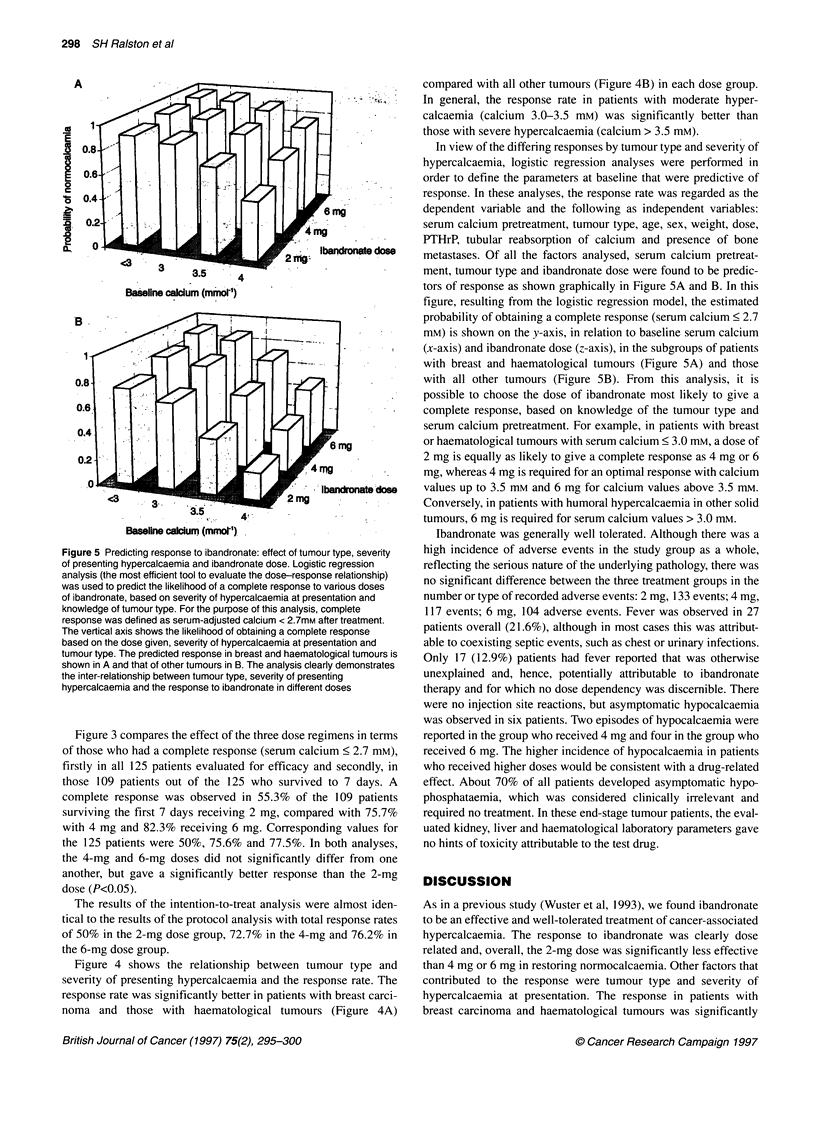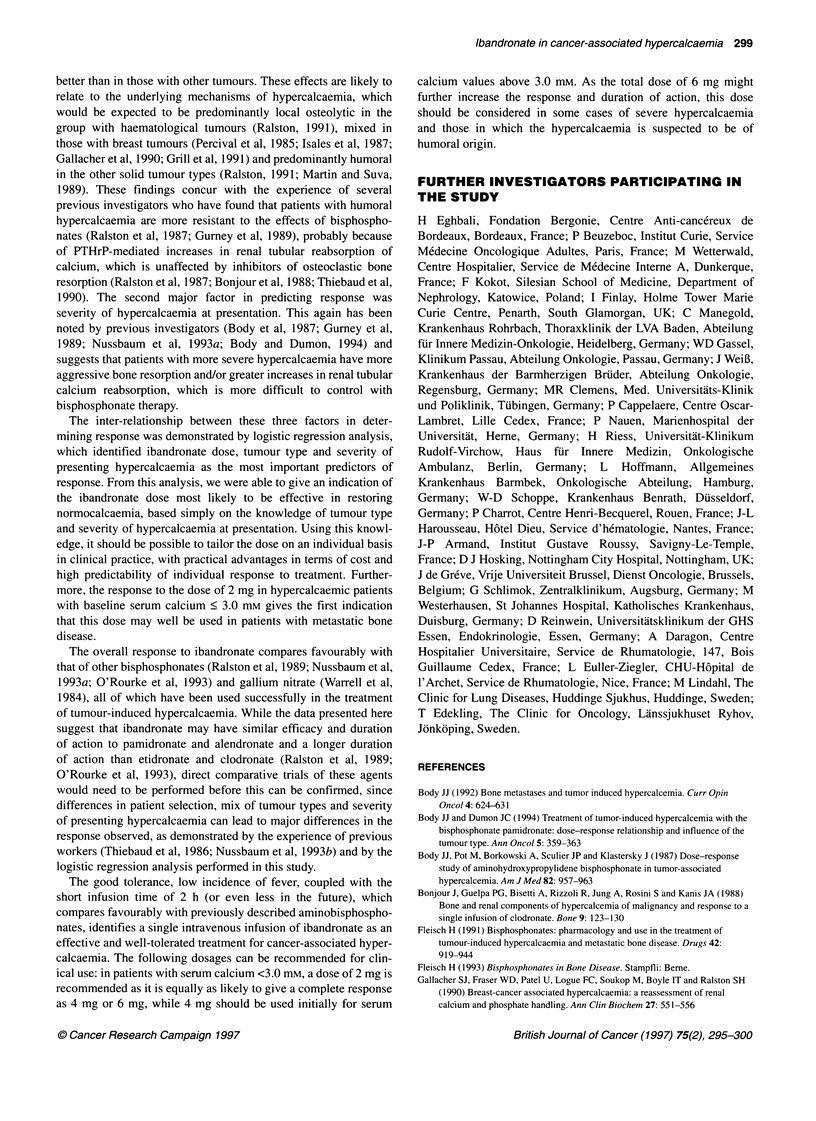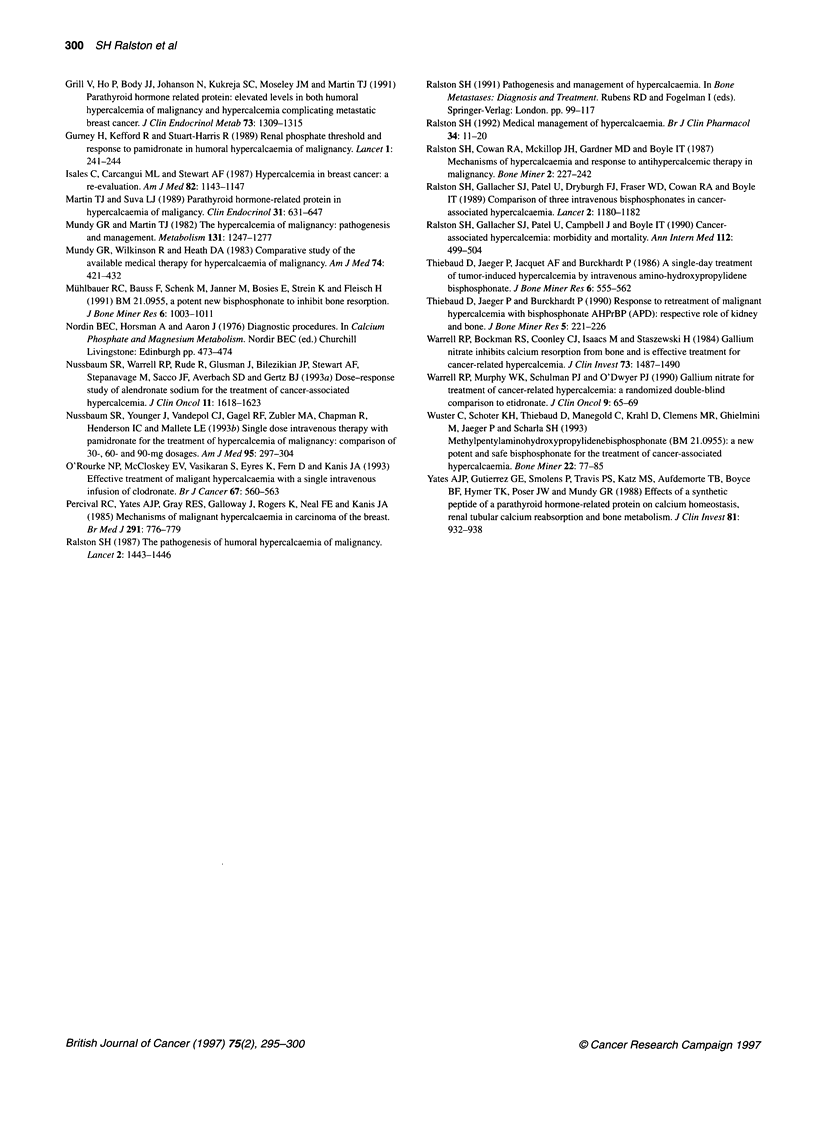# Dose-response study of ibandronate in the treatment of cancer-associated hypercalcaemia.

**DOI:** 10.1038/bjc.1997.48

**Published:** 1997

**Authors:** S. H. Ralston, D. Thiébaud, Z. Herrmann, E. U. Steinhauer, B. Thürlimann, J. Walls, M. R. Lichinitser, R. Rizzoll, H. Hagberg, H. J. Huss, M. Tubiana-Hulin, J. J. Body

**Affiliations:** University of Aberdeen, UK.

## Abstract

Hypercalcaemia is an important cause of morbidity in malignant disease. We studied the efficacy and safety of intravenous ibandronate (a new, potent bisphosphonate) in a multicentre study of 147 patients with severe cancer-associated hypercalcaemia which had been resistant to treatment with rehydration alone. Of 131 randomized patients who were eligible for evaluation, 45 were allocated to receive 2 mg ibandronate, 44 patients to receive 4 mg and 42 patients to receive 6 mg. Serum calcium values fell progressively in each group from day 2, reaching a nadir at day 5, and in some patients normocalcaemia was maintained for up to 36 days after treatment. The 2-mg dose was significantly less effective than the 4-mg or 6-mg dose in correcting hypercalcaemia, as the number of patients who achieved serum calcium values below 2.7 mM after treatment was 50% in the 2-mg group compared with 75.6% in the 4-mg group and 77.4% in the 6-mg group (P < 0.05; 2 mg vs others). In a logistic regression analysis, three factors were found to predict response; ibandronate dose (higher doses were more effective), severity of presenting hypercalcaemia (severe hypercalcaemia was associated with less complete response) and tumour type (patients with breast carcinoma and haematological tumours responded better than those with other tumours). Ibandronate was generally well tolerated and no serious drug-related adverse events were observed. We conclude that ibandronate is a safe, well tolerated and effective treatment for cancer-associated hypercalcaemia, which should prove a useful addition to the current range of therapies available to treat this condition.


British Journal of Cancer (1997) 75(2), 295–300
© 1997 Cancer Research Campaign

### Dose–response study of ibandronate in the treatment of cancer-associated hypercalcaemia

**SH Ralston[1], D Thiébaud[2], Z Herrmann[3], EU Steinhauer[4], B Thürlimann[5], J Walls[6], MR Lichinitser[7], R Rizzoli[8], H Hagberg[9], HJ Huss[3], M Tubiana-Hulin[10] and JJ Body[11]**

[1]University of Aberdeen, UK; [2]University Hospital, Lausanne, Switzerland; [3]Boehringer, Mannheim Germany; [4]City Hospital, Kassel, Germany; [5]Canton Hospital, St Gallen, Switzerland; [6]University Hospital, Manchester, UK; [7]Cancer Research Centre, Moscow, Russia; [8]University Hospital, Geneva, Switzerland; [9]University Hospital, Uppsala, Sweden; [10]Centre Rene Huguenin, Saint Cloud, France; [11]Institut Jules Bordet, Brussels

**Summary** Hypercalcaemia is an important cause of morbidity in malignant disease. We studied the efficacy and safety of intravenous ibandronate (a new, potent bisphosphonate) in a multicentre study of 147 patients with severe cancer-associated hypercalcaemia which had been resistant to treatment with rehydration alone. Of 131 randomized patients who were eligible for evaluation, 45 were allocated to receive 2 mg ibandronate, 44 patients to receive 4 mg and 42 patients to receive 6 mg. Serum calcium values fell progressively in each group from day 2, reaching a nadir at day 5, and in some patients normocalcaemia was maintained for up to 36 days after treatment. The 2-mg dose was significantly less effective than the 4-mg or 6-mg dose in correcting hypercalcaemia, as the number of patients who achieved serum calcium values below 2.7 mM after treatment was 50% in the 2-mg group compared with 75.6% in the 4-mg group and 77.4% in the 6-mg group ($P <$ 0.05; 2 mg *vs* others). In a logistic regression analysis, three factors were found to predict response; ibandronate dose (higher doses were more effective), severity of presenting hypercalcaemia (severe hypercalcaemia was associated with less complete response) and tumour type (patients with breast carcinoma and haematological tumours responded better than those with other tumours). Ibandronate was generally well tolerated and no serious drug-related adverse events were observed. We conclude that ibandronate is a safe, well tolerated and effective treatment for cancer-associated hypercalcaemia, which should prove a useful addition to the current range of therapies available to treat this condition.

**Keywords:** hypercalcaemia; parathyroid hormone-related protein; bisphosphonate; cancer; treatment

Hypercalcaemia is a common metabolic complication of malignant disease which is associated with substantial morbidity and mortality (Ralston et al, 1990). The pathophysiology of hypercalcaemia differs depending on the tumour type, but two broad categories are recognized (Mundy and Martin, 1982). In humorally mediated hypercalcaemia, the elevation in blood calcium is most often caused by release of parathyroid hormone-related protein (PTHrP), which causes a generalized increase in osteoclastic bone resorption and increased reabsorption of calcium by the renal tubule (Yates et al, 1988; Ralston, 1987; Martin and Suva, 1989). Alternatively, hypercalcaemia may arise as the result of tumour metastases in bone, which stimulate osteoclastic bone resorption on a multifocal basis, with release of calcium at a rate in excess of that which can be excreted by the kidney. In both situations, increased osteoclastic bone resorption plays an important pathogenic role, providing the rationale for treatment of cancer-associated hypercalcaemia with inhibitors of osteoclast activity (Ralston, 1992). Although several inhibitors of osteoclastic bone resorption have been used in the treatment of cancer-associated hypercalcaemia (Mundy et al, 1983; Warrell et al, 1990), bisphosphonates have emerged in recent years as a highly effective therapy, and in the view of many workers are now the treatment of first choice (Fleisch, 1991; Body, 1992; Ralston, 1992). Ibandronate (1-hydroxy-3-(methylpentyl amine) propylidene-bisphosphonate) is a new bisphosphonate that is approximately 50 times more potent than pamidronate and 500 times more potent than clodronate in inhibiting osteoclastic bone resorption in animal models (Mühlbauer et al, 1991; Fleisch, 1993). Prompted by preliminary studies which suggested that ibandronate may be of clinical value in the treatment of tumour-induced hypercalcaemia (Wuster et al, 1993), the present study was designed to assess dose–response characteristics in a double-blind randomized clinical trial.

*Received 14 May 1996*
*Revised 8 August 1996*
*Accepted 9 August 1996*

*Correspondence to:* SH Ralston, Department of Medicine and Therapeutics, University of Aberdeen AB9 2ZD, UK

### PATIENTS AND METHODS

The study was a multicentre double-blind randomized comparison of three doses of intravenous ibandronate in patients with cancer-associated hypercalcaemia. The study protocol was approved by all participating local ethics committees with the patients' written informed consent. Patients with proven malignant disease who had albumin-corrected serum calcium values of equal to or greater than 3.0 mM after a minimum of 24 h rehydration with at least 2 l of intravenous 0.9% saline or a urine output of $\geq$ 2 l day $^{-1}$ were eligible for the study. Calcium values were corrected for albumin using the formula [Serum total calcium (mM) – (0.02 × albumin (g $l^{-1}$) + 0.8)]. Patients were excluded from the study if they had evidence of significant renal impairment (serum creatinine $\geq$ 265 μM), or had other causes for the hypercalcaemia. Individuals who had been treated with bisphosphonates during the preceding

**Table 1** Pretreatment characteristics of study group

| | 2 mg ($n$=44) | 4 mg ($n$=41) | 6 mg ($n$=40) |
|---|---|---|---|
| Males ($n$/%) | 21 (47%) | 19 (46.3%) | 18 (45%) |
| Age (years) range | 58.5 (50.5–66.5) | 64 (56–68) | 56 (44.5–62) |
| Serum calcium[a] (mM) [2.2–2.7 mM] | 3.43 (3.21–3.65) | 3.38 (3.16–3.88) | 3.4 (3.19–3.63) |
| Serum phosphate (mM) [0.8–1.4 mM] | 0.88 (0.72–1.24) | 0.90 (0.78–1.2) | 0.96 (0.69–1.1) |
| Urine Ca/Cr (mM mM$^{-1}$) [<0.5] | 1.68 (1.23–2.45) | 1.76 (0.91–2.47) | 2.07 (0.89–2.55) |
| Serum creatinine (μM) [50–120 μM] | 110 (80–140) | 100 (80–140) | 110 (70–150) |
| Serum PTHrP (pM) [<2.6 pM] | 2.5 (0.9–6.6) | 3.0 (1.2–8.2) | 2.7 (1.2–6.4) |
| Total intravenous fluids given before treatment (l) | 6.0 (6.0–8.0) | 7.5 (4.6–10.0) | 7.0 (5.0–9.0) |
| Total intravenous fluids given after treatment (l) | 12.7 (5.4–44.0) | 15.0 (8.0–20.5) | 12.0 (6.0–24.0) |
| Furosemide treatment ($n$/%) | 23 (53%) | 24 (58%) | 22 (55%) |
| Tumour type | | | |
| Lung | 6 (13.6%) | 9 (21.9%) | 6 (15%) |
| Breast | 12 (27.2%) | 14 (34.1%) | 12 (30%) |
| Haematological | 2 (4.5%) | 4 (9.7%) | 6 (15%) |
| Other | 24 (54.5%) | 14 (34.1%) | 16 (40%) |
| Bone metastases | 26 (59%) | 21 (51%) | 17 (42.5%) |

Values are medians (interquartile range).[a]Albumin adjusted. Values in squared brackets show reference ranges. There was no significant difference between the groups for any variable at baseline.

**Figure 1** Response of hypercalcaemia to intravenous ibandronate. Mean ± s.e.m. of serum calcium adjusted for albumin. Significant change from day 0 is indicated by *$P$<0.05, **$P$<0.01 (Wilcoxon test). Significant difference between 2-mg *vs* 4-mg and 6-mg groups is indicated by + $P$<0.05 (Kruskal–Wallis test). Interrupted horizontal line shows upper limit of normal for adjusted calcium (2.7 mM)

3 months, plicamycin during the preceding 4 weeks and cytostatic drugs or calcitonin during the preceding week of the study were excluded. Patients who were treated with new cytostatic or anti-hypercalcaemic therapy after starting the study were censored for analysis for efficacy from that time on.

A total of 147 patients were enrolled between October 1992 and February 1994 from 36 centres in eight European countries. Fifteen patients were excluded during the run-in phase because serum calcium values fell below 3.0 mM after rehydration, or because of clinical deterioration or death. One patient who received treatment without randomization was excluded. The remaining 131 patients were randomized double blind to receive 2 mg ($n$=45), 4 mg ($n$=44) or 6 mg ($n$=42) of ibandronate by intravenous infusion over 2 h in 500 ml of intravenous saline. In order to ensure that the treatment groups were comparable in terms of tumour type and severity of hypercalcaemia, stratification for tumour type and severity of hypercalcaemia after rehydration (< 3.4 mM *vs* ≥ 3.4 mM) was carried out. Hydration (intravenous or oral) was continued after ibandronate was administered to achieve a urine output of approximately 2 l daily at the discretion of the attending physician. Treatment with loop diuretics was permitted only when clinically indicated for reasons of fluid overload or cardiac failure. Serum levels of calcium, albumin, creatinine, phosphate, alkaline phosphatase, transaminases and electrolytes were determined before treatment and at days 3 and 7 and repeated on days 14, 21 and 28. Full blood count was measured before treatment and again at days 7, 14, 21 and 28. Urinary calcium and creatinine were measured on second voided morning urine specimens, and renal tubular reabsorption was calculated (Nordin et al, 1976). Serum parathyroid hormone (PTH) and parathyroid hormone-related protein (PTHrP) concentrations were measured before treatment and at day 7 by IRMA (Nichols Institute, USA). Bone metastases were assessed by either radionuclide bone scans or plain radiographs. All biochemical and haematological investigations were performed using standard automated techniques. Patients were continuously assessed for adverse effects. The primary efficacy evaluation was based on serum-adjusted calcium levels. A complete response was defined as a serum calcium value of equal or less than 2.7 mM after treatment. Duration of action was assessed by two separate analyses: the time in normal range from response and the time to relapse, defined as an increase of albumin-corrected serum calcium to ≥ 3.0 mM in patients who had an initial response.

Statistical methods used for the evaluation of response rates were the exact test of Fisher for the comparison of treatment groups and Kaplan–Meier estimates for duration of response. Censoring was done in order not to overestimate treatment effects. Therefore, if, after response and before failure, patients died, dropped out or received i.v. bisphosphonates or calcitonin, the data of these patients were censored at that time. Patients still showing response at the end of observation were censored at their last observed trial day. The study was designed to have at least 80% power for the detection of differences ($\alpha$ < 5%) in response rates between the dose groups using a closed-test procedure. In addition, 95% confidence intervals were calculated using Pearson–Clopper values. Logistic regression analyses were performed to determine whether sex, age, weight, baseline serum calcium, tumour type, presence of bone metastases, serum PTHrP levels, tubular reabsorption of calcium and the dose of ibandronate are related to response and to evaluate dose–response surfaces, which are used for dose recommendation based on those factors significantly ($P$<0.05) contributing to response. Further exploratory analyses were performed using the Kruskal–Wallis test and the paired Wilcoxon test for comparison between and

 © Cancer Research Campaign 1997

**Figure 2** Time from response to relapse of hypercalcaemia after intravenous ibandronate. Symbols at the bottom of the graph indicate the time points in each dose group at which patients died or were censored for other reasons

**Figure 3** Dose–response to intravenous ibandronate: normalization of serum calcium. The columns show the number of patients (as a percentage) in each dose group whose serum calcium fell below 2.7 mM after treatment. The black columns refer to data from the 109 patients who survived the first 7 days and the open columns to data from all 125 patients who were evaluated for efficacy. In both instances, the response was significantly less for the 2-mg group ($P$<0.05 from other groups), but did not differ significantly between the 4-mg and 6-mg groups

**Figure 4** Response to intravenous ibandronate: effect of tumour type and severity of hypercalcaemia at presentation. The percentage of patients who showed a complete response to intravenous ibandronate (serum adjusted calcium ≤2.7 mM after treatment) is shown in relation to tumour type and severity of hypercalcaemia at presentation. The response to ibandronate tended to be better in patients with breast and haematological cancers (**A**) compared with other tumour types (B) at each ibandronate dose. For both categories of tumour type, patients with more severe hypercalcaemia (□) responded significantly less well to ibandronate than those with less severe hypercalcaemia (■) at each dose within treatment groups respectively. For all analyses, a $P$-value < 0.05 was considered as giving evidence of a significant difference. For all tests performed, a two-sided alternative was assumed.

### RESULTS

Seven patients were excluded from analysis after ibandronate administration because of protocol violations (other bisphosphonate pretreatment, serum calcium < 3.0 mM after rehydration, chemotherapy 5 days before treatment with the study drug, change in chemotherapy 3 weeks before treatment with the study drug, no sufficient follow-up data available because the patient died 1 day after the treatment, chemotherapy at start of treatment with the study drug, not randomized allocation to treatment), leaving a total of 125 patients evaluable for response. Table 1 lists the characteristics of these patients in each of the treatment groups at baseline. The three dose groups were well matched for age, sex, tumour type, presence of bone metastases, severity of hypercalcaemia and renal function as judged by serum creatinine values.

Figure 1 shows the response of serum-adjusted calcium values in the three treatment groups. Serum calcium fell in response to all three dose regimens with a nadir at day 5. The response of serum calcium was similar in patients receiving 4 mg and 6 mg, and both doses were significantly superior to the 2-mg dose. The median time in the normal range was 12 days for the 2- and 4-mg doses and 11 days for the 6-mg dose. Although the duration of the trial was specified to be 31 days, the maximum documented time in normal range lasted up to 36 days when the investigators stopped recording. More than 25% of the patients in each treatment group were in remission from hypercalcaemia at the end of the trial. Kaplan–Meier analysis (Figure 2) showed that the respective median time to relapse (defined as a rise in serum albumin-adjusted calcium to > 3.0 mM) was 18 days in the 4-mg group and 26 days in the 6-mg group, whereas in the 2-mg group more than 50% of the patients were still in remission at the end of the trial. The duration of response was not significantly different between the three treatment groups.

© Cancer Research Campaign 1997

**Figure 5** Predicting response to ibandronate: effect of tumour type, severity of presenting hypercalcaemia and ibandronate dose. Logistic regression analysis (the most efficient tool to evaluate the dose–response relationship) was used to predict the likelihood of a complete response to various doses of ibandronate, based on severity of hypercalcaemia at presentation and knowledge of tumour type. For the purpose of this analysis, complete response was defined as serum-adjusted calcium < 2.7mM after treatment. The vertical axis shows the likelihood of obtaining a complete response based on the dose given, severity of hypercalcaemia at presentation and tumour type. The predicted response in breast and haematological tumours is shown in A and that of other tumours in B. The analysis clearly demonstrates the inter-relationship between tumour type, severity of presenting hypercalcaemia and the response to ibandronate in different doses

Figure 3 compares the effect of the three dose regimens in terms of those who had a complete response (serum calcium ≤ 2.7 mM), firstly in all 125 patients evaluated for efficacy and secondly, in those 109 patients out of the 125 who survived to 7 days. A complete response was observed in 55.3% of the 109 patients surviving the first 7 days receiving 2 mg, compared with 75.7% with 4 mg and 82.3% receiving 6 mg. Corresponding values for the 125 patients were 50%, 75.6% and 77.5%. In both analyses, the 4-mg and 6-mg doses did not significantly differ from one another, but gave a significantly better response than the 2-mg dose ($P<0.05$).

The results of the intention-to-treat analysis were almost identical to the results of the protocol analysis with total response rates of 50% in the 2-mg dose group, 72.7% in the 4-mg and 76.2% in the 6-mg dose group.

Figure 4 shows the relationship between tumour type and severity of presenting hypercalcaemia and the response rate. The response rate was significantly better in patients with breast carcinoma and those with haematological tumours (Figure 4A) compared with all other tumours (Figure 4B) in each dose group. In general, the response rate in patients with moderate hypercalcaemia (calcium 3.0–3.5 mM) was significantly better than those with severe hypercalcaemia (calcium > 3.5 mM).

In view of the differing responses by tumour type and severity of hypercalcaemia, logistic regression analyses were performed in order to define the parameters at baseline that were predictive of response. In these analyses, the response rate was regarded as the dependent variable and the following as independent variables: serum calcium pretreatment, tumour type, age, sex, weight, dose, PTHrP, tubular reabsorption of calcium and presence of bone metastases. Of all the factors analysed, serum calcium pretreatment, tumour type and ibandronate dose were found to be predictors of response as shown graphically in Figure 5A and B. In this figure, resulting from the logistic regression model, the estimated probability of obtaining a complete response (serum calcium ≤ 2.7 mM) is shown on the $y$-axis, in relation to baseline serum calcium ($x$-axis) and ibandronate dose ($z$-axis), in the subgroups of patients with breast and haematological tumours (Figure 5A) and those with all other tumours (Figure 5B). From this analysis, it is possible to choose the dose of ibandronate most likely to give a complete response, based on knowledge of the tumour type and serum calcium pretreatment. For example, in patients with breast or haematological tumours with serum calcium ≤ 3.0 mM, a dose of 2 mg is equally as likely to give a complete response as 4 mg or 6 mg, whereas 4 mg is required for an optimal response with calcium values up to 3.5 mM and 6 mg for calcium values above 3.5 mM. Conversely, in patients with humoral hypercalcaemia in other solid tumours, 6 mg is required for serum calcium values > 3.0 mM.

Ibandronate was generally well tolerated. Although there was a high incidence of adverse events in the study group as a whole, reflecting the serious nature of the underlying pathology, there was no significant difference between the three treatment groups in the number or type of recorded adverse events: 2 mg, 133 events; 4 mg, 117 events; 6 mg, 104 adverse events. Fever was observed in 27 patients overall (21.6%), although in most cases this was attributable to coexisting septic events, such as chest or urinary infections. Only 17 (12.9%) patients had fever reported that was otherwise unexplained and, hence, potentially attributable to ibandronate therapy and for which no dose dependency was discernible. There were no injection site reactions, but asymptomatic hypocalcaemia was observed in six patients. Two episodes of hypocalcaemia were reported in the group who received 4 mg and four in the group who received 6 mg. The higher incidence of hypocalcaemia in patients who received higher doses would be consistent with a drug-related effect. About 70% of all patients developed asymptomatic hypophosphataemia, which was considered clinically irrelevant and required no treatment. In these end-stage tumour patients, the evaluated kidney, liver and haematological laboratory parameters gave no hints of toxicity attributable to the test drug.

### DISCUSSION

As in a previous study (Wuster et al, 1993), we found ibandronate to be an effective and well-tolerated treatment of cancer-associated hypercalcaemia. The response to ibandronate was clearly dose related and, overall, the 2-mg dose was significantly less effective than 4 mg or 6 mg in restoring normocalcaemia. Other factors that contributed to the response were tumour type and severity of hypercalcaemia at presentation. The response in patients with breast carcinoma and haematological tumours was significantly

*© Cancer Research Campaign 1997*

better than in those with other tumours. These effects are likely to relate to the underlying mechanisms of hypercalcaemia, which would be expected to be predominantly local osteolytic in the group with haematological tumours (Ralston, 1991), mixed in those with breast tumours (Percival et al, 1985; Isales et al, 1987; Gallacher et al, 1990; Grill et al, 1991) and predominantly humoral in the other solid tumour types (Ralston, 1991; Martin and Suva, 1989). These findings concur with the experience of several previous investigators who have found that patients with humoral hypercalcaemia are more resistant to the effects of bisphosphonates (Ralston et al, 1987; Gurney et al, 1989), probably because of PTHrP-mediated increases in renal tubular reabsorption of calcium, which is unaffected by inhibitors of osteoclastic bone resorption (Ralston et al, 1987; Bonjour et al, 1988; Thiebaud et al, 1990). The second major factor in predicting response was severity of hypercalcaemia at presentation. This again has been noted by previous investigators (Body et al, 1987; Gurney et al, 1989; Nussbaum et al, 1993*a*; Body and Dumon, 1994) and suggests that patients with more severe hypercalcaemia have more aggressive bone resorption and/or greater increases in renal tubular calcium reabsorption, which is more difficult to control with bisphosphonate therapy.

The inter-relationship between these three factors in determining response was demonstrated by logistic regression analysis, which identified ibandronate dose, tumour type and severity of presenting hypercalcaemia as the most important predictors of response. From this analysis, we were able to give an indication of the ibandronate dose most likely to be effective in restoring normocalcaemia, based simply on the knowledge of tumour type and severity of hypercalcaemia at presentation. Using this knowledge, it should be possible to tailor the dose on an individual basis in clinical practice, with practical advantages in terms of cost and high predictability of individual response to treatment. Furthermore, the response to the dose of 2 mg in hypercalcaemic patients with baseline serum calcium ≤ 3.0 mM gives the first indication that this dose may well be used in patients with metastatic bone disease.

The overall response to ibandronate compares favourably with that of other bisphosphonates (Ralston et al, 1989; Nussbaum et al, 1993*a*; O'Rourke et al, 1993) and gallium nitrate (Warrell et al, 1984), all of which have been used successfully in the treatment of tumour-induced hypercalcaemia. While the data presented here suggest that ibandronate may have similar efficacy and duration of action to pamidronate and alendronate and a longer duration of action than etidronate and clodronate (Ralston et al, 1989; O'Rourke et al, 1993), direct comparative trials of these agents would need to be performed before this can be confirmed, since differences in patient selection, mix of tumour types and severity of presenting hypercalcaemia can lead to major differences in the response observed, as demonstrated by the experience of previous workers (Thiebaud et al, 1986; Nussbaum et al, 1993*b*) and by the logistic regression analysis performed in this study.

The good tolerance, low incidence of fever, coupled with the short infusion time of 2 h (or even less in the future), which compares favourably with previously described aminobisphosphonates, identifies a single intravenous infusion of ibandronate as an effective and well-tolerated treatment for cancer-associated hypercalcaemia. The following dosages can be recommended for clinical use: in patients with serum calcium <3.0 mM, a dose of 2 mg is recommended as it is equally as likely to give a complete response as 4 mg or 6 mg, while 4 mg should be used initially for serum calcium values above 3.0 mM. As the total dose of 6 mg might further increase the response and duration of action, this dose should be considered in some cases of severe hypercalcaemia and those in which the hypercalcaemia is suspected to be of humoral origin.

### FURTHER INVESTIGATORS PARTICIPATING IN THE STUDY

H Eghbali, Fondation Bergonie, Centre Anti-cancéreux de Bordeaux, Bordeaux, France; P Beuzeboc, Institut Curie, Service Médecine Oncologique Adultes, Paris, France; M Wetterwald, Centre Hospitalier, Service de Médecine Interne A, Dunkerque, France; F Kokot, Silesian School of Medicine, Department of Nephrology, Katowice, Poland; I Finlay, Holme Tower Marie Curie Centre, Penarth, South Glamorgan, UK; C Manegold, Krankenhaus Rohrbach, Thoraxklinik der LVA Baden, Abteilung für Innere Medizin-Onkologie, Heidelberg, Germany; WD Gassel, Klinikum Passau, Abteilung Onkologie, Passau, Germany; J Weiß, Krankenhaus der Barmherzigen Brüder, Abteilung Onkologie, Regensburg, Germany; MR Clemens, Med. Universitäts-Klinik und Poliklinik, Tübingen, Germany; P Cappelaere, Centre Oscar-Lambret, Lille Cedex, France; P Nauen, Marienhospital der Universität, Herne, Germany; H Riess, Universität-Klinikum Rudolf-Virchow, Haus für Innere Medizin, Onkologische Ambulanz, Berlin, Germany; L Hoffmann, Allgemeines Krankenhaus Barmbek, Onkologische Abteilung, Hamburg, Germany; W-D Schoppe, Krankenhaus Benrath, Düsseldorf, Germany; P Charrot, Centre Henri-Becquerel, Rouen, France; J-L Harousseau, Hôtel Dieu, Service d'hématologie, Nantes, France; J-P Armand, Institut Gustave Roussy, Savigny-Le-Temple, France; D J Hosking, Nottingham City Hospital, Nottingham, UK; J de Gréve, Vrije Universiteit Brussel, Dienst Oncologie, Brussels, Belgium; G Schlimok, Zentralklinikum, Augsburg, Germany; M Westerhausen, St Johannes Hospital, Katholisches Krankenhaus, Duisburg, Germany; D Reinwein, Universitätsklinikum der GHS Essen, Endokrinologie, Essen, Germany; A Daragon, Centre Hospitalier Universitaire, Service de Rhumatologie, 147, Bois Guillaume Cedex, France; L Euller-Ziegler, CHU-Hôpital de l'Archet, Service de Rhumatologie, Nice, France; M Lindahl, The Clinic for Lung Diseases, Huddinge Sjukhus, Huddinge, Sweden; T Edekling, The Clinic for Oncology, Länssjukhuset Ryhov, Jönköping, Sweden.

### REFERENCES

Body JJ (1992) Bone metastases and tumor induced hypercalcemia. *Curr Opin Oncol* **4**: 624–631

Body JJ and Dumon JC (1994) Treatment of tumor-induced hypercalcemia with the bisphosphonate pamidronate: dose–response relationship and influence of the tumour type. *Ann Oncol* **5**: 359–363

Body JJ, Pot M, Borkowski A, Sculier JP and Klastersky J (1987) Dose–response study of aminohydroxypropylidene bisphosphonate in tumor-associated hypercalcemia. *Am J Med* **82**: 957–963

Bonjour J, Guelpa PG, Bisetti A, Rizzoli R, Jung A, Rosini S and Kanis JA (1988) Bone and renal components of hypercalcemia of malignancy and response to a single infusion of clodronate. *Bone* **9**: 123–130

Fleisch H (1991) Bisphosphonates: pharmacology and use in the treatment of tumour-induced hypercalcaemia and metastatic bone disease. *Drugs* **42**: 919–944

Fleisch H (1993) *Bisphosphonates in Bone Disease*. Stampfli: Berne.

Gallacher SJ, Fraser WD, Patel U, Logue FC, Soukop M, Boyle IT and Ralston SH (1990) Breast-cancer associated hypercalcaemia: a reassessment of renal calcium and phosphate handling. *Ann Clin Biochem* **27**: 551–556

© Cancer Research Campaign 1997 

Grill V, Ho P, Body JJ, Johanson N, Kukreja SC, Moseley JM and Martin TJ (1991) Parathyroid hormone related protein: elevated levels in both humoral hypercalcemia of malignancy and hypercalcemia complicating metastatic breast cancer. *J Clin Endocrinol Metab* **73**: 1309–1315

Gurney H, Kefford R and Stuart-Harris R (1989) Renal phosphate threshold and response to pamidronate in humoral hypercalcaemia of malignancy. *Lancet* **1**: 241–244

Isales C, Carcangui ML and Stewart AF (1987) Hypercalcemia in breast cancer: a re-evaluation. *Am J Med* **82**: 1143–1147

Martin TJ and Suva LJ (1989) Parathyroid hormone-related protein in hypercalcaemia of maligancy. *Clin Endocrinol* **31**: 631–647

Mundy GR and Martin TJ (1982) The hypercalcemia of malignancy: pathogenesis and management. *Metabolism* **131**: 1247–1277

Mundy GR, Wilkinson R and Heath DA (1983) Comparative study of the available medical therapy for hypercalcaemia of malignancy. *Am J Med* **74**: 421–432

Mühlbauer RC, Bauss F, Schenk M, Janner M, Bosies E, Strein K and Fleisch H (1991) BM 21.0955, a potent new bisphosphonate to inhibit bone resorption. *J Bone Miner Res* **6**: 1003–1011

Nordin BEC, Horsman A and Aaron J (1976) Diagnostic procedures. In *Calcium Phosphate and Magnesium Metabolism.* Nordir BEC (ed.) Churchill Livingstone: Edinburgh pp. 473–474

Nussbaum SR, Warrell RP, Rude R, Glusman J, Bilezikian JP, Stewart AF, Stepanavage M, Sacco JF, Averbach SD and Gertz BJ (1993*a*) Dose–response study of alendronate sodium for the treatment of cancer-associated hypercalcemia. *J Clin Oncol* **11**: 1618–1623

Nussbaum SR, Younger J, Vandepol CJ, Gagel RF, Zubler MA, Chapman R, Henderson IC and Mallete LE (1993*b*) Single dose intravenous therapy with pamidronate for the treatment of hypercalcemia of malignancy: comparison of 30-, 60- and 90-mg dosages. *Am J Med* **95**: 297–304

O'Rourke NP, McCloskey EV, Vasikaran S, Eyres K, Fern D and Kanis JA (1993) Effective treatment of maligant hypercalcaemia with a single intravenous infusion of clodronate. *Br J Cancer* **67**: 560–563

Percival RC, Yates AJP, Gray RES, Galloway J, Rogers K, Neal FE and Kanis JA (1985) Mechanisms of malignant hypercalcaemia in carcinoma of the breast. *Br Med J* **291**: 776–779

Ralston SH (1987) The pathogenesis of humoral hypercalcaemia of malignancy. *Lancet* **2**: 1443–1446

Ralston SH (1991) Pathogenesis and management of hypercalcaemia. In *Bone Metastases: Diagnosis and Treatment.* Rubens RD and Fogelman I (eds). Springer-Verlag: London. pp. 99–117

Ralston SH (1992) Medical management of hypercalcaemia. *Br J Clin Pharmacol* **34**: 11–20

Ralston SH, Cowan RA, Mckillop JH, Gardner MD and Boyle IT (1987) Mechanisms of hypercalcaemia and response to antihypercalcemic therapy in malignancy. *Bone Miner* **2**: 227–242

Ralston SH, Gallacher SJ, Patel U, Dryburgh FJ, Fraser WD, Cowan RA and Boyle IT (1989) Comparison of three intravenous bisphosphonates in cancer-associated hypercalcaemia. *Lancet* **2**: 1180–1182

Ralston SH, Gallacher SJ, Patel U, Campbell J and Boyle IT (1990) Cancer-associated hypercalcemia: morbidity and mortality. *Ann Intern Med* **112**: 499–504

Thiebaud D, Jaeger P, Jacquet AF and Burckhardt P (1986) A single-day treatment of tumor-induced hypercalcemia by intravenous amino-hydroxypropylidene bisphosphonate. *J Bone Miner Res* **6**: 555–562

Thiebaud D, Jaeger P and Burckhardt P (1990) Response to retreatment of malignant hypercalcemia with bisphosphonate AHPrBP (APD): respective role of kidney and bone. *J Bone Miner Res* **5**: 221–226

Warrell RP, Bockman RS, Coonley CJ, Isaacs M and Staszewski H (1984) Gallium nitrate inhibits calcium resorption from bone and is effective treatment for cancer-related hypercalcemia. *J Clin Invest* **73**: 1487–1490

Warrell RP, Murphy WK, Schulman PJ and O'Dwyer PJ (1990) Gallium nitrate for treatment of cancer-related hypercalcemia: a randomized double-blind comparison to etidronate. *J Clin Oncol* **9**: 65–69

Wuster C, Schoter KH, Thiebaud D, Manegold C, Krahl D, Clemens MR, Ghielmini M, Jaeger P and Scharla SH (1993) Methylpentylaminohydroxypropylidenebisphosphonate (BM 21.0955): a new potent and safe bisphosphonate for the treatment of cancer-associated hypercalcaemia. *Bone Miner* **22**: 77–85

Yates AJP, Gutierrez GE, Smolens P, Travis PS, Katz MS, Aufdemorte TB, Boyce BF, Hymer TK, Poser JW and Mundy GR (1988) Effects of a synthetic peptide of a parathyroid hormone-related protein on calcium homeostasis, renal tubular calcium reabsorption and bone metabolism. *J Clin Invest* **81**: 932–938

© Cancer Research Campaign 1997